# Disability and morbidity among US birth cohorts, 1998–2018: A multidimensional test of dynamic equilibrium theory

**DOI:** 10.1016/j.ssmph.2023.101528

**Published:** 2023-10-04

**Authors:** Tianyu Shen, Collin F. Payne

**Affiliations:** a School of Demography, Australian National University, Canberra, Australia; b Harvard Center for Population and Development Studies, Harvard T.H. Chan School of Public Health, Harvard University, Boston, MA, USA

**Keywords:** Morbidity, Disability, Aging, Dynamic equilibrium, Health expectancy

## Abstract

A substantial body of prior research has explored patterns of disability-free and morbidity-free life expectancy among older populations. However, these distinct facets of later-life health are almost always studied in isolation, even though they are very likely to be related. Using data from the US Health and Retirement Study and a multistate life table approach, this paper explores the interactions between disability, morbidity, and mortality by sex and education among four successive US birth cohorts, born from 1914 to 1923 to 1944–1953 and compared in the periods 1998–2008 and 2008–2018. We find little compression of disability but a marked expansion of morbidity across cohorts. However, disability-free life expectancy (DFLE) among those living with chronic morbidities has increased, even though at the population-level DFLE is largely unchanged. Broadly, these patterns suggest that successive cohorts of older populations in the US are experiencing a dynamic equilibrium, where the link between chronic morbidities and disability has weakened over successive cohorts. Investigating patterns by educational attainment, we find marked disparities where the least educated individuals not only live significantly fewer years free of disabilities or chronic morbidities but also have experienced an expansion in morbidity and disability. Our findings suggest that the future trajectory of disability-free life expectancy in the US is increasingly contingent on efforts to improve disease management and control the severe consequences of chronic morbidities.

## Highlights

• Successive cohorts in the US population are living more years with chronic diseases.
• Life expectancy with disability has not changed substantially over cohorts.
• Educational inequality in morbidity- and disability-free life is substantial.
• Healthy longevity is increasingly contingent on chronic disease management.

## 1 Introduction

Notwithstanding a recent period of stagnation and decline prior to and during the COVID-19 pandemic, life expectancy in the United States both at birth and age 65 has grown almost linearly over the past decades (National Center for Health Statistics, 2021). Americans alive in 2019 can expect to live nearly five years longer than the average person in their parents’ generation 30–40 years earlier, and almost a decade longer than the average person alive in the 1950s. Although these increases in longevity are undoubtedly an applaudable achievement, there is considerable debate on whether these gains in life expectancy are being matched by increases in life with good health.

Previous studies present consistent evidence of the contrasting trends in different facets of health over time in the recent US population. On the one hand, analyses looking at morbidity, as defined by the presence of a set of chronic health conditions, generally find that an expansion of morbidity is happening in the US population over time (e.g., Beltrán-Sánchez et al., 2015; Crimmins & Beltrán-Sánchez, 2011; Payne, 2022). Crimmins et al. (2019) suggest that the prevalence of cancer, stroke and diabetes has increased over successive 10-year cohorts, but that mortality associated with these diseases has declined significantly. On the other hand, recent research on trends in disability in the US finds that the proportion of life with disability has been rather stable over time (Bardenheier et al., 2016; Crimmins, 2015; Payne, 2022).

A limitation of the existing evidence base on patterns of healthy longevity is that most existing studies explore variation in later-life well-being on only a single axis—that is, most studies look at functional limitation/disability, chronic morbidities, self-rated health, or other domains of health in isolation. In this paper, we propose a multidimensional extension to prior work on health expectancy by simultaneously modelling changes in morbidity and disability across a set of cohorts in the US population to better understand changes over time/cohort and interactions between morbidity and disability. This multidimensional approach allows us to test for another less discussed theory: dynamic equilibrium theory (Manton, 1982), which emphasizes how morbidity, and the effect of morbidities on disability, may change over time and across cohorts. Apart from examining patterns in males and females, we utilize this approach to explore the heterogeneities in health by educational subgroups, exploring how educational disparities may arise in the interactions between morbidity, disability, and mortality.

The terms “morbidity” and “disability” could be overlapping depending on their definitions. In the expansion/compression of morbidity theories (Fries, 1980; Gruenberg, 1977), “morbidity” also refers to “disability”, while, in sociology, “disability” may include chronic illness (Mauldin & Brown, 2021). In this paper, we limit our definition of morbidity to the presence of major chronic diseases, while the effect of morbidity is conceptualized as the presence of limitations in activities of daily living (ADLs) based on the pathway described in the disablement process by Verbrugge and Jette (1994). Although morbidity and disability are highly interconnected markers of health status, they represent distinct dimensions of individuals’ health. On the one hand, ADL disability could result from accidents, mental or cognitive health issues, or from other limitations in living conditions that may not be directly attributable to chronic disease. On the other hand, morbidity may not result in physical limitation if these morbidities are diagnosed early and well controlled. Exploring these facets of health jointly provides a more comprehensive marker of health than exploring either in isolation.

## 2 Theoretical framework

Two conceptual frameworks have predominated discussions of trends in health in later life: the expansion of morbidity framework of Gruenberg (1977) and the compression of morbidity theory, promoted by Fries (1980, 2005). Considerable debate still exists as to whether recent expansions in lifespan are being met, or exceeded, by an expansion of time spent healthy. Fries’ conceptual framework positing a compression of morbidity relied heavily on an assumed strong linkage between chronic morbidities, disability, and mortality: “Disability and lowered quality of life due to the most prevalent chronic diseases are thus unescapably linked with eventual mortality” (Fries, 1980, p. 132). As pointed out by Beltrán-Sánchez et al. (2014), most work investigating the compression of morbidity has followed Fries assumed strong connection between morbidity and disability and focused on disability as the primary outcome of interest.

The dynamic equilibrium model developed by Manton (1982) takes a different approach to understanding population health change over time and across cohorts. A key tenet of this model is that over time, advances in medical technology, treatment, and early diagnoses may weaken the linkage between chronic morbidities and disability. In essence, the dynamic equilibrium model suggests that different facets of population health may move in different directions over time: as life expectancy increases, the fraction of remaining life spent with chronic morbidities may increase (due to improved diagnoses of diseases at early stages and improved treatment effectiveness prolonging lifespan), while simultaneously the fraction of remaining life spent with functional disability may stay constant or decline (due to better management of these chronic morbidities). However, little research has explicitly tested whether the patterns predicted by dynamic equilibrium theory are occurring in the US population.

Our focus on exploring the interplay between these two facets of health draws on both the disablement process model of Verbrugge and Jette (1994) and the dynamic equilibrium theory of Manton (1982). In the disablement process, the main path of disability onset connects physiological dysregulation to functional impairments/limitations. In addition, both the disablement process and the dynamic equilibrium model recognize that linkages between health conditions and functional deficits are malleable, unlike the deterministic morbidity-disability linkage posited by Fries. Guided by these frameworks, this research aims to jointly analyze connections between morbidity, disability, and mortality to better understand the relationship and transitions between these dimensions of health.

Social inequalities in the US population are also likely to lead to heterogeneity in the dynamic pathways connecting chronic morbidities, disability, and mortality. Prior research on healthy longevity has highlighted the considerable social inequalities existing in the US population by race, level of education, and occupational status (Chiu et al., 2016; Solé-Auró et al., 2015; Zaninotto, Batty, et al., 2020). Among these sociodemographic characteristics, educational attainment has become an increasingly important factor stratifying mortality and health in later life (Sasson, 2016). Additionally, the role of educational inequalities in shaping healthy longevity is likely multifaceted—that is, the combination of stress processes, material deprivation, and unequal access to and treatment by the healthcare system experienced by lower educated groups is likely to have an impact on different pathways of the disablement process. Education could theoretically moderate the pathway between chronic diseases and disability through interactions with different risk factor exposures and social environment experienced over the life course (Zajacova & Lawrence, 2018). However, prior research has not explicitly sought to understand how connections between chronic morbidities, disability, and mortality stratify by education groups, nor has it explored how these connections may change over time.

Our analytical approach centers on measuring changes in healthy longevity from a cohort perspective. This approach builds on both foundational work on the connections between period and cohort measures of disability-free life expectancy (DFLE) (e.g., Manton et al., 2008; Manton & Land, 2000; Soneji, 2006) and recent work exploring cohort changes in disability, chronic diseases, and health in the United States (e.g., Beltrán-Sánchez et al., 2016; Crimmins et al., 2019; Payne, 2022). A cohort perspective on healthy longevity more closely represents the lived experience of individuals within the population, allowing us to measure whether a change in healthy longevity (i.e., a compression of morbidity) is being experienced by an actual group of individuals in the population. Taking a cohort perspective on social inequalities in healthy longevity may also produce different results as compared to a period perspective. Beltrán-Sánchez et al. (2015) found that the period-based studies produce more consistent results in terms of the disparities by sex, race, and education, while cohort studies usually give mixed results on the inequality across groups. They attribute these contradictory findings to the methodological differences between period and cohort — period measures rely on synthetic cohort approaches that combine data across numerous cohorts, aggregating age-specific death rates and age-specific morbidity/disability conditions for that period. As such, a period-based analysis does not represent the real-life experience of any individual or social group within the population because it aggregates many cohorts across a wide range of ages. The downside to focusing on cohort measures of later life health is that we cannot measure full-cohort life and health expectancies—that is, total remaining life expectancy, and expectancies of life lived with/without disability or morbidity. Instead, our analysis focuses on comparing partial LE and health expectancies within bounded age-ranges.

### 2.1 Research hypotheses

Our study aims to explore cohort trends in health, testing whether patterns in the US population align with the three theories discussed in the last section. If an average individual spends more time with morbidity and disability over cohorts, these patterns suggest an expansion of morbidity (*Hypothesis 1*). Conversely, if an average individual spends less time with morbidity and disability across cohorts, these patterns suggest a compression of morbidity (*Hypothesis 2*).

Empirical support for dynamic equilibrium theory is manifested in a growing discrepancy between cohort changes in morbidity and disability. In the case where the time spent with morbidity for an average individual increases, while the time with disability hardly changes or declines slightly, the patterns would support dynamic equilibrium theory (*Hypothesis 3*). However, another important criterion of dynamic equilibrium theory is the reduction in disease severity over time. To operationalize this change, we need to rely on the connected pathway from physiological dysregulation to functional disability in the disablement process. In other words, functional disability is one of the overt outcomes of the progression of disease. Thus, in the case of dynamic equilibrium, an average individual with chronic morbidities should spend more time disability-free across successive cohorts, due to a reduction in disease severity (*Hypothesis 4*). Only when both *Hypothesis 3* (spending more time with morbidity but not spending more time with disability across cohorts) and *Hypothesis 4* (spending more time without disability if morbid) are satisfied do the patterns suggest alignment with dynamic equilibrium theory.

Our analysis centers on three objectives. First, we explore how patterns of life expectancy with chronic morbidities and functional disability have changed over successive cohorts in the US population and test *Hypotheses 1*, *2* and *3*. Second, we use a multidimensional approach to investigate how connections between chronic morbidities and disability have changed over cohorts, testing *Hypothesis 4*. Lastly, to further understand health inequality among population sub-groups, we investigate whether there is heterogeneity in these patterns by educational attainment and whether different educational groups align with the same theory. We employ the same multidimensional test to examine these same four hypotheses for each educational group. The results offer an in-depth understanding of educational disparities in the pathways connecting these different facets of health, and how they have changed across successive cohorts.

## 3 Data & methods

### 3.1 Data

Data are from the US Health and Retirement Survey (HRS), a bi-annual national longitudinal survey (Sonnega et al., 2014). Our analysis uses the RAND HRS Longitudinal File 2018 (V1) developed at RAND with funding from the National Institute on Aging and the Social Security Administration (Health and Retirement Study, 2021). We use data from the 1998 to 2018 waves of data collection, focusing on individuals aged 60 and above to compare the change in time spent with disability and/or morbidity between consecutive cohorts. Table 1 presents the four birth cohorts used in the analysis highlighted in different colors (also see Supplemental Fig. S1 for a Lexis diagram presenting the cohort comparison for the youngest age group). Depending on the period (year) and age that the cohort is observed, the four birth cohorts are labelled as “early” or “later” cohorts in the figures according to Table 1.

**Table 1:** Age, period, and birth cohort comparisons.

Period Observed	1998–2008	2008–2018
Age Range		
60–69	1934–1943	1944–1953
70–79	1924–1933	1934–1943
80–89	1914–1923	1924–1933

### 3.2 Measures

Two dimensions of health are measured: morbidity and disability. Morbidity is measured by five variables in the survey inquiring “whether the respondent has ever been diagnosed by the physician with ___ (a chronic disease)”. We focus on the five top causes of death from chronic non-communicable diseases in the US (Kochanek et al., 2020): cancer (excluding minor skin cancers), diabetes, heart disease (combining prior heart attack, coronary heart disease, angina, and congestive heart failure), lung disease (combining chronic bronchitis and emphysema) and stroke. Defining morbidity as ever-diagnosed is a widely used approach in health research (e.g., Chatterji et al., 2015; Gu et al., 2009; Payne, 2022; Zaninotto, Head, & Steptoe, 2020). Individuals with these chronic conditions are likely to rely on some forms of treatment to control disease progression for the rest of their lives. Individuals without any of the five chronic diseases are classified as “morbidity-free (MF)” and “morbid (M)” otherwise.

Disability is conceptualized as reporting difficulty in doing basic daily activities on the Activities of Daily Living (ADL) scale (Katz et al., 1963): bathing, dressing, eating, transferring in/out of bed, and walking across a room. Individuals reporting no limitations are classified as “disability-free (DF)”, those reporting one or more ADL limitations are classified as “disabled (D)”. Where necessary, ADL status from a proxy respondent is used.

Morbidity and disability are combined into one measure with 4 combinations of health status: morbidity-free & disability-free (MF-DF), morbidity-free & ADL disabled (MF-D), morbid & disability-free (M-DF) and morbid & ADL disabled (M-D). The Incidence of death is captured through linkages to the National Death Index (NDI) as well as exit interviews with the respondent's family members (Sonnega et al., 2014). The age of death is calculated from the date of death variable in the interview.

Covariates in the analysis include age as a continuous variable, sex as a dichotomous variable (female and male), and a 10-year birth cohort as a categorical variable with four consecutive cohorts: 1914–1923, 1924–1933, 1934–1943, and 1944–1953. For the education model, a covariate for level of attained education is included with three categories: 1) less than high school diploma, 2) high school graduate (including GED), and 3) above high school diploma (including some college and bachelor's degree or higher). Baseline characteristics in the sample by age group and birth cohort are summarized in Table 2. The baseline of birth cohort 1934–1943 in 1998 includes respondents from 55 to 64 years old, for example (also see Fig. S1).

**Table 2:** Sample characteristics of the birth cohorts by age at baseline.

Age	60		70		80	
Birth Cohort	1934–43Early	1944–53	1924–33	1934–43	1914–23	1924–33
	Early	Later	Early	Later	Early	Later
N	7114	4451	5389	5790	3410	3593
	(%)	(%)	(%)	(%)	(%)	(%)
*Sex*						
Men	47.6	47.7	44.7	46.4	40.0	40.7
Women	52.4	52.3	55.3	53.6	60.0	59.3
*Race/ethnicity*						
White	80.8	78.1	83.2	80.3	87.3	83.3
Black	9.7	10.2	9.1	9.1	7.1	8.2
Hispanic	7.4	8.5	5.5	8.1	4.1	6.5
Other	2.1	3.2	2.2	2.4	1.5	2.0
*Educational* attainment						
<HS	20.8	11.0	28.4	20.2	33.0	25.5
HS grad	37.2	31.7	36.0	36.8	36.8	37.2
>HS	42.0	57.3	35.6	43.0	30.2	37.3
*1* + *ADL-disabled*	10.8	10.8	13.7	13.7	22.0	23.7
*1* + *Morbidity*	30.6	37.5	45.9	53.4	57.1	65.0
*State*						
MF-DF	64.5	58.5	49.8	42.9	36.4	29.7
MF-D	5.0	4.0	4.3	3.7	6.5	5.2
M-DF	24.7	30.8	36.5	43.4	41.6	46.6
M-D	5.8	6.7	9.4	10.0	15.5	18.5

### 3.3 Methods

**Fig. 1:**
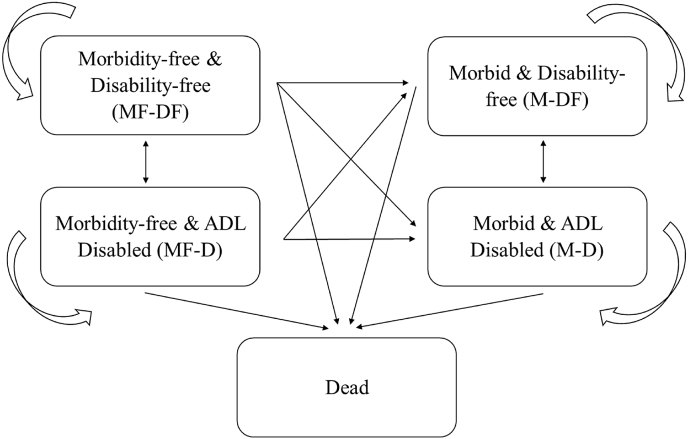
State-space and transition relationships between health states.

We apply a five-state multistate life table model to estimate population-based partial cohort life expectancy (PC-LE) and partial cohort health expectancies (PC-HE). The transitions among these five states are depicted in Fig. 1. Transitions from Morbid (M) to Morbidity-free (MF) are not allowed since the definition of morbidity in our analysis is “ever diagnosed”. To estimate transition probabilities, we convert the survey data from two-year intervals to single-year intervals, randomly assigning the missing state to either the last observed state or the next observed state (Payne, 2022). We obtain the exact age of death from the HRS data, and individuals who die between survey waves are assumed to stay in their last observed state until death.

Two multinomial regression models are built to model the annual transition probabilities with different covariates as analogous to Cai et al. (2010). Different cohorts are pooled together to estimate the transition probability of each age group. For example, transition probabilities in ages 60–69 are estimated from ages 55–74 for cohort 1934–1943 in 1998–2008 and cohort 1944–1953 in 2008–2018. The first multinomial model is the population-level analysis with age, age-squared, sex, birth cohorts and interactions between age, sex, and birth cohorts. The second one explores educational disparity by including all covariates in the population-level model, as well as level of attained education, and two interaction terms between level of attained education and sex or birth cohort. All the coefficients in these two models are statistically significant and improve model fit.

Two types of weights are used in the regressions, sample weight and attrition weight. The sample weight is available in the HRS dataset (combined respondent weight and nursing home resident weight). The attrition weight is used to account for potential bias resulting from differential loss to follow-up. We generate and use an inverse probability weight to rescale individuals who do not attrit according to their age, sociodemographic characteristics and health status as shown in Table 2 (Dugoff et al., 2014; Payne, 2022). The final weights are the product of sample weights and attrition weights.

We use the modelled coefficient to generate the transition probabilities for each group of people by age. These transition probabilities are then used as inputs for a microsimulation-based multistate life table model (Cai et al., 2010). To estimate population-based LE, we generate a synthetic cohort of 100,000 individuals for each age and cohort group in Table 1 with sociodemographic and initial health distribution as in the observed data (sample characteristics in Table 2 with sample weights). These individuals are aged year by year using age- and sex-specific estimated transition probabilities between different states. The process is repeated from the starting age to the ending age for each model, age group, and birth cohort. For example, we generate the synthetic cohort of 100,000 individuals with the same baseline characteristics of the 1934–1943 birth cohort and simulate life-courses of these individuals from age 60 to 69 based on the transition probabilities described above. The average time spent in different states over these ten years is our estimate of PC-HE. Only the point estimates from the multinomial regression models are used; confidence intervals (CIs) are estimated by bootstrap resampling from the original dataset. We re-estimate and simulate based on these 500 bootstrap samples. The final point estimates presented in the results are from the full dataset, and the central 95% of the 500 bootstrap resamples times is taken as the 95% CI. Our analysis is conducted in R software (R Core Team, 2023).

## 4 Results

The sample characteristics of each cohort by age at baseline are presented in Table 2. Educational attainment is much higher in the later cohorts as compared to the early ones, and within each age group, successive cohorts are slightly more diverse in terms of race/ethnicity. The proportions of people with one or more chronic diseases (M-DF + M-D) increases markedly over successive cohorts. There is little change to the proportion of each cohort reporting limitations on ADL activities, but there is a small, consistent drop in the proportion of ADL disabled individuals without chronic diseases and a rise in ADL disabled individuals with chronic diseases.

### 4.1 Health expectancies by sex

**Fig. 2:**
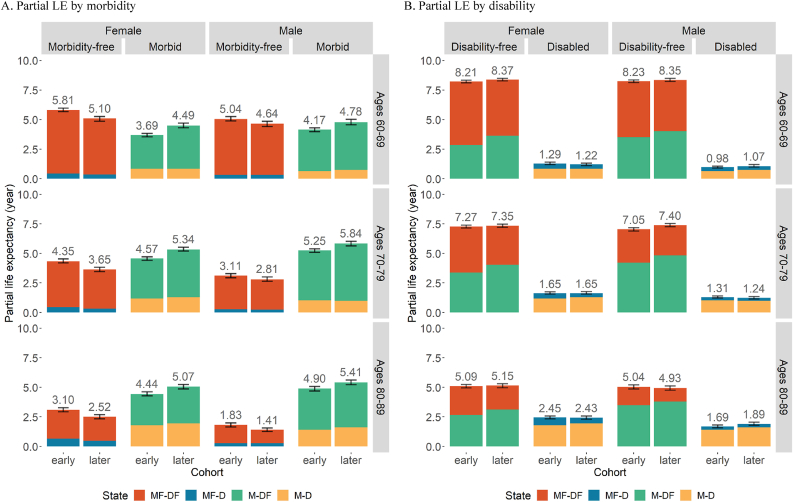
Estimated PC-HE across birth cohorts with 95% CI. *Notes*: The figure above each bar shows the total partial LE of that bar and the error bar is the 95% CI for that total partial LE. Values in each state can also be found in Appendix Table S3. *Source*: As for Table 2

**Fig. 3:**
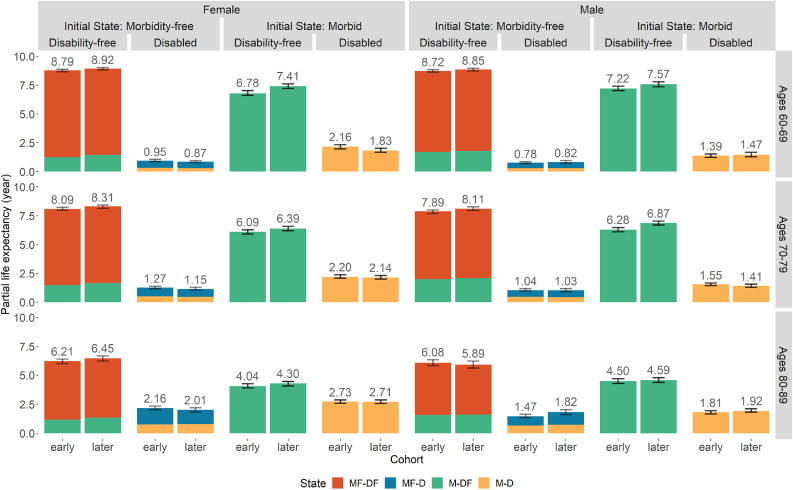
Estimated PC-HE across birth cohorts by initial state of morbidity with 95% CI. *Notes*: The figure above each bar shows the total partial LE of that bar and the error bar is the 95% CI for that total partial LE. *Source*: As for Table 2.

**Fig. 4:**
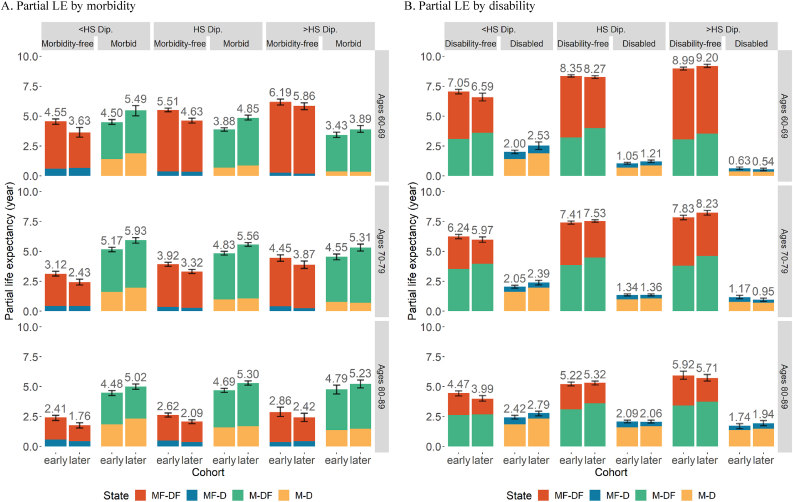
Estimated PC-HE across birth cohorts by educational attainment with 95% CI. *Notes*: The figure above each bar shows the total partial LE of that bar and the error bar is the 95% CI for that total partial LE. Values in each state can also be found in Appendix Table S4. *Source*: As for Table 2

The estimated PC-HEs in each state in our analysis are presented by sex (Fig. 2, Fig. 3 & Supplemental Fig. S2). The bars are the aggregated average time spent in each health status for an individual with those characteristics or initial health state. In Fig. 2, Fig. 4, there are two panels: A) by morbidity and B) by disability. Panel A of Fig. 2 shows the time without (MF) and with chronic diseases (M) combined across disability status, while Panel B illustrates the time without (DF) and with disability (D) combined across morbidity status. Note that the PC-HE estimates in these two panels are identical—the only difference is how they are grouped. Fig. 3 additionally disaggregates by initial morbidity state to more clearly identify into how disability-free and disabled life expectancies have changed across cohorts. Supplemental Fig. S2 further disaggregates by all initial states. The sum of MF & M or DF & D in each age range and cohort for all the figures is the total PC-LE of that group of individuals. For example, the partial cohort morbidity-free LE (PC-MFLE) of female in Panel A of Fig. 2 at age 60–69 (the top left section) is 5.81 years for the early cohort (1934–1943) and partial morbid life expectancy (PC-MLE) is 3.69 years, and hence the total PC-LE is 9.50 years. The corresponding section in Panel B shows partial disability-free life expectancy (PC-DFLE) at 8.21 years and partial disabled life expectancy (PC-DLE) at 1.29 years, which also sums to total PC-LE of 9.50 years (also found in Table S1). The “early” and “later” cohorts in the figures correspond to different cohorts that are presented in Table 1. Furthermore, PC-LE has increased marginally over cohorts in each age range.

#### 4.1.1 Morbidity

Across all age ranges in our study, life expectancy spent free of chronic morbidities has declined across successive cohorts, and life expectancy with one or more chronic diseases has climbed significantly (Fig. 2 Panel A). Put another way, the total PC-LE has increased for all age ranges, but PC-MFLE has decreased significantly. In general, females spent more years of life morbidity-free than their male counterparts. This gender gap in PC-MFLE widens as people grow older—by ages 80–89, an average female in the early cohort (1914–1923) could expect to spend 3.10 (95% CI: 2.93, 3.27) years free of chronic morbidities, compared with only 1.83 years (95% CI: 1.65, 2.00) for a male. However, the gap appears to be smaller in the later cohorts for all ages. Focusing on the morbidity-free column, the red color represents LE spent morbidity-free and disability-free (MF-DF) while the blue color represents LE spent morbidity-free and disabled (MF-D). For both males and females, time spent free of disability and morbidity (i.e., PC-MDFLE) decreased significantly over successive cohorts in all age groups (also see Table S1).

Turning to LE with chronic morbidities (the “Morbid” column of Panel A), we find that partial cohort morbid LE (PC-MLE) rises significantly across cohorts for both males and females. Since PC-MLEs has increased across all age ranges and sex groups, these patterns agree with either *Hypothesis 1* or *3*. Most of this change can be attributed to an increase in disability-free life with chronic morbidities (denoted by the green bars, also see Panel B and Table S1). Comparing the green bar across the early and later cohorts demonstrates that partial life expectancy spent with chronic morbidities but without disability (M-DF) has risen significantly across cohorts—in ages 60–69, females in the more recently-born cohort (1944–1953) can expect to spend an additional 0.79 years with chronic morbidities, but without disability, as compared to those in the early cohort (1934–1943), while this figure is 0.52 for males. We also find gender differences in these cohort patterns. Males spend significantly fewer years morbid and disabled (M-D), suggesting that males are more likely to stay disability-free while having chronic diseases than women.

#### 4.1.2 Disability

Panel B of Fig. 2 provides partial cohort DFLE (“Disability-free” column) and DLE (“Disabled” column) by sex. Overall, males spend roughly the same time disability-free as females, but spend fewer years disabled, leading to the gap in total PC-LE between males and females. The PC-DFLE and PC-DLE remain largely unchanged across cohorts, though we do see consistent, although mostly marginal, rises in PC-DFLE across cohorts. In successive cohorts, the portion of life expectancy spent disability-free is increasingly made up of years with chronic diseases—for both males and females. The proportion of disability-free life spent with chronic diseases increased. Similarly, the proportion of PC-DLE spent with chronic morbidities also increased across cohorts, though to a lesser extent. These trends are supportive of *Hypothesis 3* (or dynamic equilibrium theory), because the time spent in disability is not growing in parallel with the observed increases in time spent with morbidities.

#### 4.1.3 Disability by morbidity status

In Fig. 3, the PC-HEs are further disaggregated by the initial morbidity state to account for the changes in baseline composition across cohorts (as shown in Table 2). There is again a consistent but minor rise across cohorts in PC-DFLE for individuals free of chronic diseases at the state of each age range (Initial State: Morbidity-free). The increase in PC-DFLE is much larger for individuals starting morbid (Initial State: Morbid), particularly in ages 60–69 and 70–79. PC-DFLE is higher for males who initially had chronic morbidities than for their female counterparts after the disaggregation by initial state. This improvement in disability-free life among those with chronic morbidities is more evident in panel B of Supplemental Fig. S2 where all initial states are separated. PC-DFLE has increased significantly for individuals with chronic morbidities at ages 60 and 70 (M-DF or M-D). In ages 80–89 PC-DFLE is also trending upwards over cohorts, although the improvements are relatively small (particularly for males). This increase in PC-DFLE for morbid individuals across cohorts largely supports our *Hypothesis 4*. These results suggest that cohort improvements in overall PC-DFLE may have been stopped by the growing proportion of individuals who had chronic morbidities at baseline (as shown in Table 2) even though we find significant improvement in PC-DFLE of morbid individuals. Combining with the patterns found in the previous sections, the changes in population health by sex across cohorts predominantly align with dynamic equilibrium theory.

As discussed above, time spent free of both disability and chronic morbidities (PC-MDFLE) declined significantly across cohorts for both sexes (Fig. 2). Females' PC-MDFLE is consistently higher than males’ for all age ranges. However, time spent with both disability and chronic morbidities remained stable; the loss in PC-MDFLE has been offset by a rise in life expectancy with chronic morbidities but without disability. Furthermore, after controlling for initial morbidity state (Fig. 3), time spent morbidity- and disability-free (red bar) is almost unchanged across cohorts. Again, these patterns suggest that trends in the US population are following those predicted by dynamic equilibrium theory.

### 4.2 Educational differences in health expectancies

Fig. 3, Fig. 4, and Supplemental Fig. S3, present our results separately by highest level of schooling. An education gradient is evident in both morbidity and disability, as well as mortality. PC-MFLE and PC-DFLE of the most educated individuals (beyond high school diploma) is significantly higher than that of the least educated ones (those without a high school diploma) for almost all age ranges and cohorts (Fig. 4). Likewise, time spent without disability and chronic morbidities (PC-MDFLE) and the PC-LE are significantly greater for the most educated individuals (see Table S2).

#### 4.2.1 Morbidity

In all educational groups, both PC-MFLE and PC-MDFLE decrease across cohorts for all age ranges, while PC-MLE increased (Panel A of Fig. 4). However, the magnitude of these increases varied—increases in LE with chronic morbidities are smallest for the highest educated group and largest among those with less than a high school diploma (see Table S2). Among time spent with chronic morbidities, those in the lowest schooling group could expect to spend four to five times (depending on cohort) more years with ADL disability (yellow bar) as compared to those with beyond a high school diploma in ages 60–69, although this gap shrinks with increasing age. The significant increases across cohorts in PC-MLE found in most age and educational groups corroborate either *Hypothesis 1* or *3*.

#### 4.2.2 Disability

Trends in PC-DLE by level of schooling (Panel B of Fig. 4) show that contrasting trends are occurring among the lowest- and highest-educated groups. Successive cohorts of individuals without a high school diploma experience expansions of time spent with disability. In addition, the PC-DLE for individuals with a high school diploma at ages 60–69 also increases significantly across cohorts. For these less educated individuals, their cohort changes, combined with the findings in the last section, support *Hypothesis 1*: the expansion of morbidity. In contrast, those with a high school diploma or above are living approximately the same amount or slightly less time with disability across successive cohorts. These trends are more aligned with *Hypothesis 3* and satisfy the initial criteria for dynamic equilibrium theory. The highest educated group at ages 80–89 are somewhat of an exception to these trends, as both PC-DLE and PC-MLE have increased marginally. These patterns appear to align closer with *Hypothesis 1* but given the small size and selectivity of this population these results are somewhat difficult to interpret.

#### 4.2.3 Disability by morbidity status

**Fig. 5:**
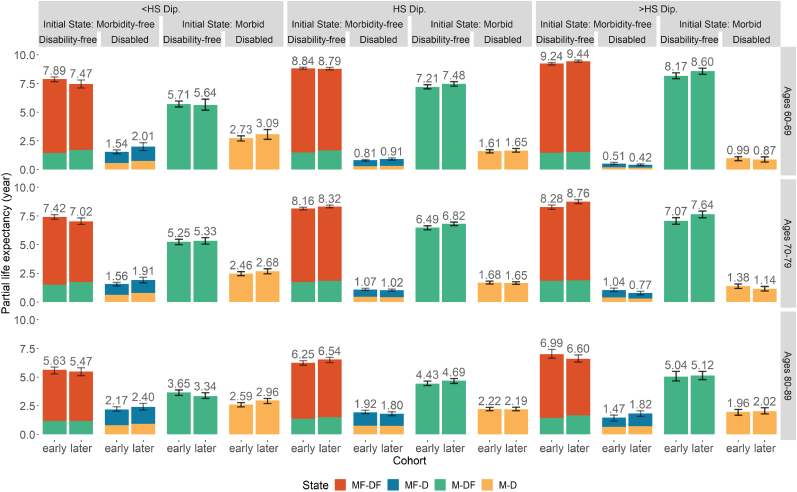
Estimated PC-HE across birth cohorts by educational attainment and initial state of morbidity with 95% CI. *Notes*: The figure above each bar shows the total partial LE of that bar and the error bar is the 95% CI for that total partial LE. *Source*: As for Table 2

Individuals are separated by their initial state in Fig. 5 and Supplemental Fig. S3 to further investigate *Hypothesis 4* and disparities between levels of education. The patterns of PC-DFLE appear to diverge by educational attainment in Fig. 5. For the lowest educated group, individuals who are initially morbidity-free can expect to spend less time in disability-free for most ages, while those with initial morbidities experience little change in PC-DFLE, which aligns with *Hypothesis 1*.

For those in higher education groups, an upward trend in PC-DFLE over cohorts is evident, conditional on initial morbidity status. Furthermore, the more detailed disaggregation in Supplemental Fig. S3 demonstrates a significant expansion in PC-DFLE in most age-ranges for the more educated group (high school or above) for those with initial morbidities but without disability (M-DF). At ages 80–89, there is little expansion or compression of morbidity and disability across cohorts for most subpopulations after controlling for the initial state. This finding likely stems from the relatively low life expectancies of these groups of older individuals, which leaves less time for differences between subpopulations to arise. In short, our results broadly support *Hypothesis 4*, and we find that cohort trends in health of the more educated groups fit best with dynamic equilibrium theory. However, we find distinct patterns for those without a high school diploma, where our results align better with the expansion of morbidity theory.

## 5 Discussion

This study evaluates cohort patterns in time spent in disability and/or morbidity in the US by sex and level of education. We identify a few key findings on cohort trends in health from our results. Firstly, consistent with evidence from the Human Mortality Database (2023) on cohort life expectancy, PC-LE increases across successive cohorts, with females living longer than males at all age ranges. Secondly, life expectancy spent with chronic morbidities (PC-MLE) has expanded over cohorts, especially in younger age ranges. PC-MFLE of females is much higher compared to their male counterparts. Thirdly, PC-DFLE and PC-DLE are largely stable across cohorts. However, PC-DFLE has increased significantly among individuals with one or more morbidities, even though the overall PC-DFLE has stayed largely unchanged. Lastly, time spent free of both chronic morbidities and disability declined significantly across cohorts for both sexes. Again, females' PC-MDFLE is consistently higher than males’ for all age ranges. Nevertheless, PC-MDFLE remains largely unchanged across cohorts when controlling for initial state. Overall, these findings suggest that the cohort trends by sex align the best with dynamic equilibrium theory.

Beyond the population level, we find notable heterogeneity in PC-LE and PC-HEs by level of education. More advantaged subpopulations with higher educational attainment spend more years without disabilities and/or morbidities and longer total life expectancy. Apart from these advantages at the overall level, these subpopulations are also likely to experience improvements in PC-DFLE if they start morbid. These disparities can be most easily spotted between the lowest education group and the highest education groups. Successive cohorts of lower-schooled individuals have experienced an expansion of disability and morbidity, while the more educated groups have only experienced a significant expansion of morbidity, but little change in disability. Furthermore, the PC-DFLE of individuals with at least high school diploma increased across cohorts for those starting with morbidities. In other words, we find that different theories of morbidity change are applicable to cohort trends depending on level of educational attainment: the lowest educated individuals have seen an expansion of morbidity, whereas the cohort changes for those with high school diploma or more are experiencing a dynamic equilibrium.

We find that increases in life expectancy with morbidity are ubiquitous across the birth cohorts and groups in this paper. At the same time, average duration with disability is not increasing in tandem with this expansion of time spent with morbidity for most groups. These trends appear to contradict either the compression of morbidity speculated by (Fries, 1980, 2005) or the expansion of morbidity and disability predicted by (Gruenberg, 1977). Our results demonstrate that the overarching trend in population health among older Americans is towards an increase in disability-free life years lived with chronic diseases. Although we observe a decrease in average time spent in disability across successive cohorts for individuals with chronic morbidities, the overall life expectancy free from disabilities has not improved. Despite the significant expansion in DFLE for people with chronic morbidities, DFLE is still much higher for individuals without chronic morbidities. Said another way, the expanding prevalence of chronic morbidities at the population level appears to be an important factor impeding the compression of disability.

Advancements in treatment to control the symptoms and progression of diseases may have played a major role in producing these findings (Crimmins, 2015). These trends are likely the result of two interrelated and ongoing processes: i) advancement in medicine, which can control the progression of chronic disease, and ii) earlier diagnoses of chronic conditions at less severe stages. Broadly constructed, we provide evidence that successive cohorts of the US population are experiencing patterns that most closely align with Manton's Dynamic Equilibrium framework (Manton, 1982), where life expectancy improvements may have resulted from reductions in the severity and rate of progression of chronic diseases. However, the heterogeneities found among education groups imply that these reductions in the rate of progression of chronic diseases are not equal across the population. As suggested by prior literature (Chang & Lauderdale, 2009), we theorize that less educated individuals may be less likely to receive timely benefits from medical advances that could improve management of chronic health conditions and delay disability. These disparities are not likely the direct result of educational attainment alone, but rather are more likely derived from a mixture of disadvantages experienced by individuals and embedded within the broader socioeconomic context (Zajacova & Lawrence, 2018).

Our findings also demonstrate the value of looking beyond a purely disability-based framework for understanding healthy longevity, as suggested by Beltrán-Sánchez et al. (2014). Across cohorts, we find declines in lifetime spent free of both chronic morbidities and disability, which may translate to rising costs of healthcare, medications, and disability services. These patterns are particularly salient given the age compositional changes occurring in the US population as the baby boom cohorts enter later life. Current projections estimate that the costs of Medicare-funded health services will rise to nearly 6% of the US gross domestic product by 2040, up from just under 4% in 2020—a difference largely driven by overall population aging (House of Representatives Congress, 2020). However, these projections do not account for changing cohort patterns of morbidity, which could further inflate these costs. Thus, the focus of public health, and public policy, should not be only on reducing the consequences of diseases but also on delaying the onset of chronic diseases (Beltrán-Sánchez et al., 2015). At the individual level, the financial and social costs resulting from a longer time spent with chronic diseases may also explain some of the disparities by level of education, as inequality in access to medicines and services could restrict individuals’ ability to control the progression, and limit the disabling impacts, of chronic diseases.

Several limitations should be considered when interpreting our results. Firstly, the MSLT model used is a Markov model, which assumes that the immediate future state depends solely on the current state and not on the past health trajectory. While common in MSLT method, this assumption may overlook unobserved effects from earlier states. Another limitation results from the biannual nature of the HRS, assuming only one transition between waves. However, actual individuals might face back-and-forth transitions within two years and potential health deterioration before death. Additionally, our analysis uses partial cohort measures of healthy longevity. This approach is useful for direct application of the results because it examines the actual lived experience of a group of individuals in a population (e.g., Beltrán-Sánchez et al., 2016; Manton et al., 2008; Payne, 2022). Despite its benefits, these partial cohort estimates may not always align with completed cohort measures, particularly in cases of rapid advancements in disease treatment (Payne & Kobayashi, 2022). Therefore, these partial cohort estimates should not replace full cohort estimates. Lastly, the definition of morbidity in this study relies on a somewhat blunt measure of ever-diagnosed with any of the five chronic diseases. Grouping individuals with different disease severities together as morbid may lead to an increased prevalence of morbid individuals across cohorts partly due to earlier disease detection. HRS data are not well suited to comprehensively assessing disease severity, and future research is needed to explore health trends accounting for the disease severity.

Future research could potentially extend this paper in several ways. Firstly, the microsimulation approach employed here offers valuable insights into the synthetic life course, allowing exploration of additional quantities, like average ages of transition, beyond life and health expectancies available from classic MSLT methods. Secondly, while this paper focuses on educational inequality, there are other pertinent personal and environmental factors proposed by Verbrugge and Jette (1994) that merit consideration and incorporation into the estimation, such as racial disparities and differences between native-born Americans and various migrant groups. However, these analyses would involve minority groups that may require incorporating data beyond the HRS. Thirdly, this study measures morbidity using a binary variable, but prior research (Quiñones et al., 2016) suggests that different comorbidity combinations may pose distinct risks for disablement. However, incorporating such nuances into existing multistate life table models is challenging, as increasing the number of health dimensions or categories of measurement greatly increases the state space of the model, quickly leading to estimation issues. Last, health expectancies are determined by both the initial population structure and the observed schedule of transition probabilities. As mentioned above, the potential compression in disability may be counteracted by expansion in the prevalence of morbidity at the initial age. Therefore, it could be interesting and informative to use a decomposition approach to determine the source of change across cohorts (Shen et al., 2023).

## 6 Conclusion

In conclusion, this study tests dynamic equilibrium theory empirically, filling a knowledge gap on how chronic morbidities and disability interact across successive cohorts in the US population. Our findings reveal that, for most subpopulations (especially younger ages), partial cohort morbid life expectancy has increased across all age groups. However, partial cohort DFLE and DLE remain remarkably stable over cohorts. This relative stability in disability-free life is underpinned by marked changes in the population prevalence of chronic morbidities: DFLE has increased significantly for individuals with chronic morbidities. These patterns align closely with the dynamic equilibrium theory. Examining heterogeneity by educational levels, we, however, find that successive cohorts without a high school diploma can expect to spend increasing time with chronic morbidities and disability. Considering the large increase in time spent with chronic morbidities, our findings suggest that the future trajectory of disability-free life expectancy in the US is increasingly contingent on efforts to improve disease management and control the severe consequences of chronic morbidities.

## Ethical statement

No ethical approval was required as this is a secondary analysis of data gathered by third parties.

## Funding

This work was supported by a Discovery Early Career Researcher Award (DE210100087) funded by the 10.13039/501100000923Australian Research Council and also by an ANU Futures Scheme Award funded by the 10.13039/501100000995Australian National University.

## Financial disclosure statement

These funding body played no role in the design, collection, analysis and interpretation of the data.

## Author statement

Tianyu Shen: Conceptualization, Methodology, Data analysis, Writing - Original Draft, Writing - Review & Editing.

Collin F. Payne: Conceptualization, Writing – Review & Editing, Funding acquisition.

## Declaration of competing interest

The authors declare that they have no known competing financial interests or personal relationships that could have appeared to influence the work reported in this paper.

## Data Availability

The data can be obtained from the Health and Retirement Study, https://hrsdata.isr.umich.edu/data-products/rand
