# Supplementary material for: Disability and morbidity among US birth cohorts, 1998–2018: A multidimensional test of dynamic equilibrium theory
Source: SSM Popul Health. 2023 Oct 4;24:101528. doi: 10.1016/j.ssmph.2023.101528 (PMC10625143; doi:10.1016/j.ssmph.2023.101528)
Supplement: Multimedia component 1 [file mmc1.docx]

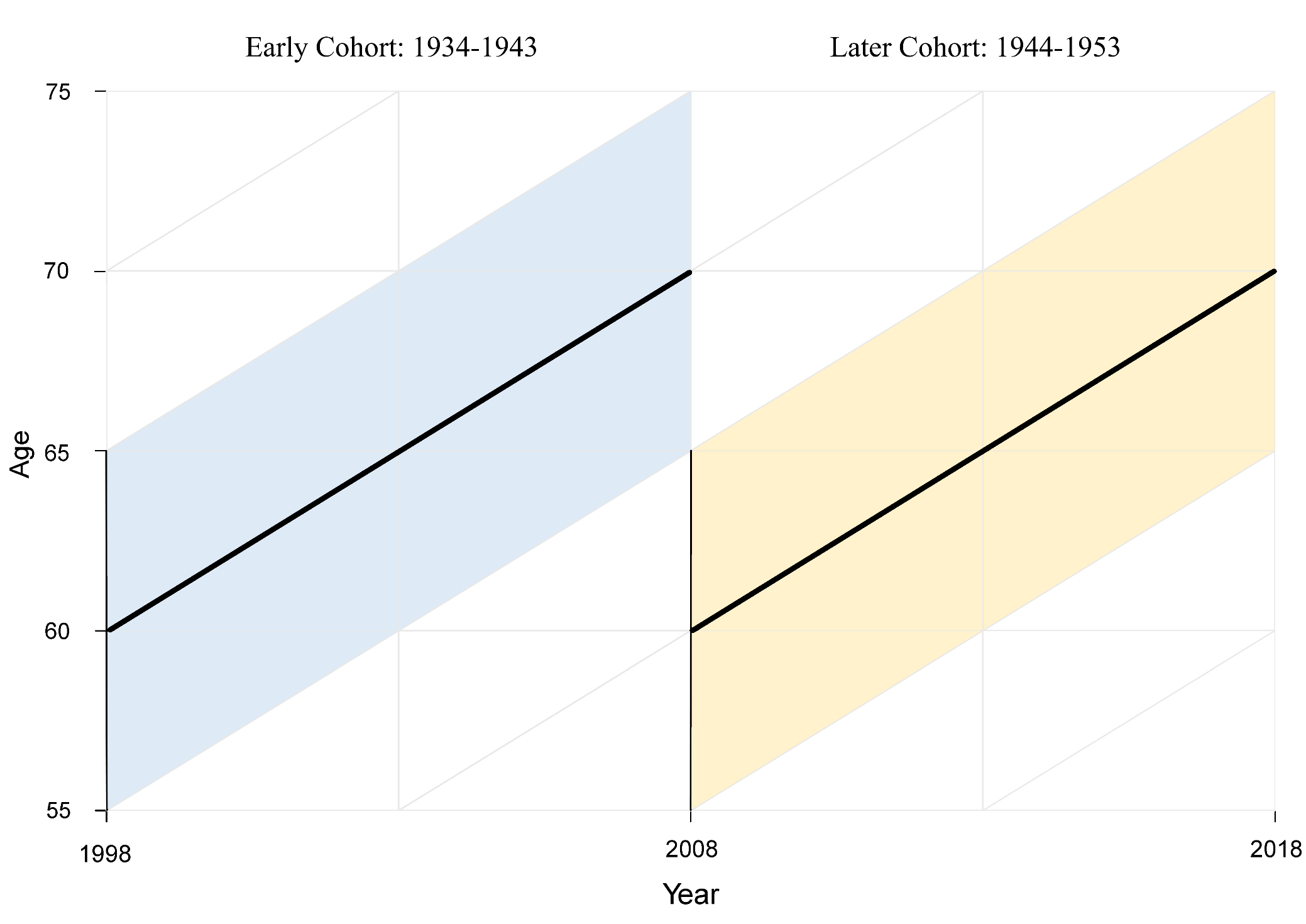


**Figure S1**. Lexis diagram of birth cohorts 1934-1943 and 1944-1953


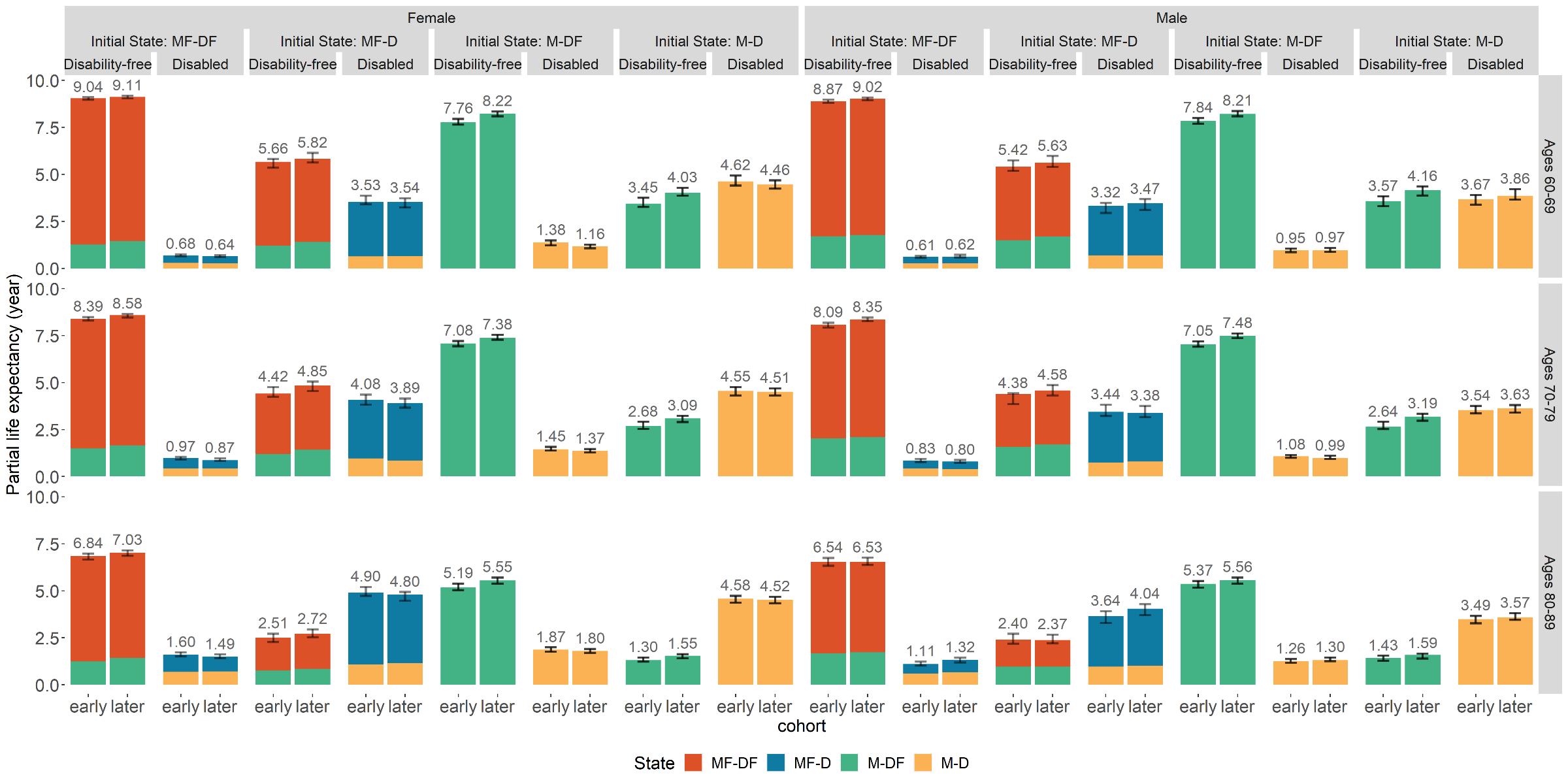


**Figure S2**. Status-based health expectancy by sex with 95% CI. *Notes*: The figure above each bar shows the total partial LE of that bar and the error bar is the 95% CI for that total partial LE.


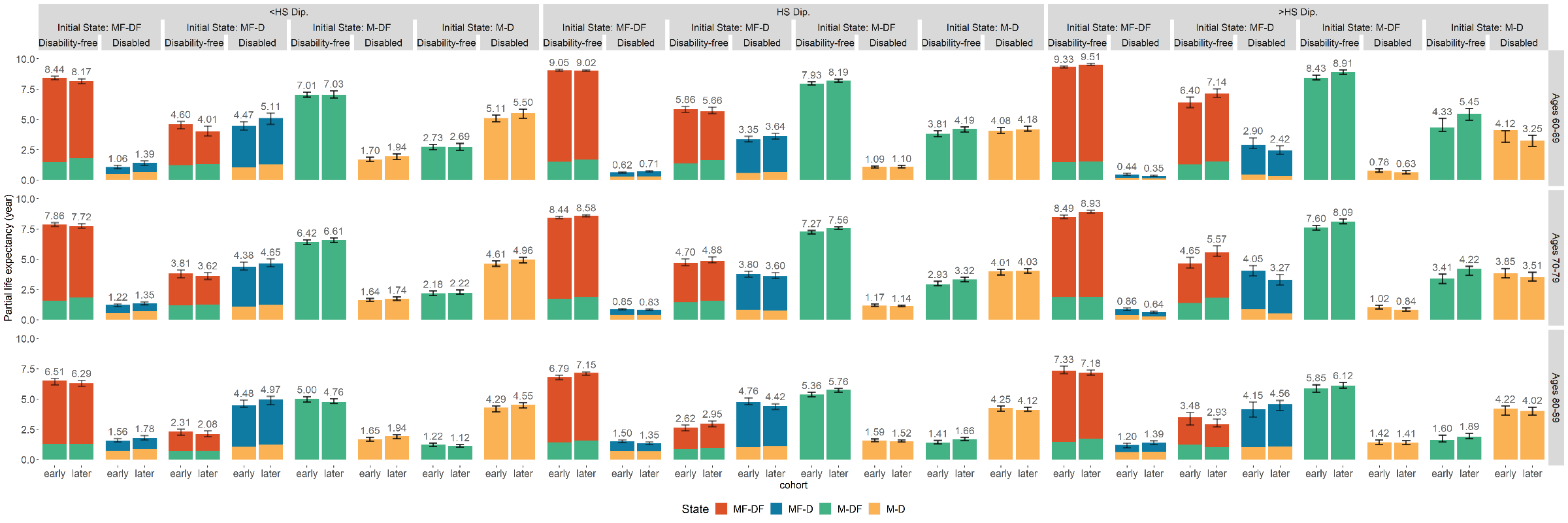


**Figure S3**. Status-based health expectancy by educational attainment with 95% CI. *Notes*: The figure above each bar shows the total partial LE of that bar and the error bar is the 95% CI for that total partial LE.

**Table S1.** Partial cohort health expectancies by sex

| **Ages** |  | **Early Cohort** | | | | **Later Cohort** | | | |
| --- | --- | --- | --- | --- | --- | --- | --- | --- | --- |
| **60-69** | **Cohort**  **Sex** | 1934-1943 | | | | 1944-1953 | | | |
|  | **Female** |  | Morbidity-free **(MF)** | Morbid **(M)** | **Total** |  | Morbidity-free **(MF)** | Morbid **(M)** | **Total** |
|  |  | Disability-free **(DF)** | 5.37  (5.21, 5.52) | 2.84  (2.71, 2.98) | **8.21**  **(8.10,8.32)** | Disability-free **(DF)** | 4.74  (4.52, 4.93) | 3.63  (3.47, 3.85) | **8.37**  **(8.27, 8.48)** |
|  |  | Disabled **(D)** | 0.45  (0.40, 0.52) | 0.85  (0.77, 0.92) | **1.29**  **(1.21, 1.41)** | Disabled **(D)** | 0.36  (0.30, 0.41) | 0.86  (0.78, 0.94) | **1.22**  **(1.12. 1.31)** |
|  |  | **Total** | **5.81**  **(5.68, 5.98)** | **3.69**  **(3.53, 3.84)** | **9.50**  **(9.46, 9.56)** | **Total** | **5.10**  **(4.87, 5.27)** | **4.49**  **(4.31, 4.71)** | **9.59**  **(9.54, 9.64)** |
|  | **Male** |  | **MF** | **M** | **Total** |  | **MF** | **M** | **Total** |
|  |  | **DF** | 4.72  (4.57, 4.93) | 3.51  (3.36, 3.65) | **8.23**  **(8.13, 8.36)** | **DF** | 4.33  (4.09, 4.54) | 4.03  (3.82, 4.24) | **8.35**  **(8.22, 8.48)** |
|  |  | **D** | 0.32  (0.28, 0.37) | 0.66  (0.58, 0.72) | **0.98**  **(0.90, 1.06)** | **D** | 0.32  (0.26, 0.39) | 0.75  (0.67, 0.86) | **1.07**  **(0.98, 1.21)** |
|  |  | **Total** | **5.04**  **(4.89, 5.25)** | **4.17**  **(3.98, 4.31)** | **9.21**  **(9.15, 9.28)** | **Total** | **4.64**  **(4.42, 4.86)** | **4.78**  **(4.57, 5.03)** | **9.43**  **(9.37, 9.50)** |
| **70-79** | **Cohort**  **Sex** | 1924-1933 | | | | 1934-1943 | | | |
|  | **Female** |  | **MF** | **M** | **Total** |  | **MF** | **M** | **Total** |
|  |  | **DF** | 3.89  (3.75, 4.05) | 3.38  (3.24, 3.51) | **7.27**  **(7.14, 7.39)** | **DF** | 3.31  (3.13, 3.46) | 4.04  (3.87, 4.20) | **7.35**  **(7.20, 7.48)** |
|  |  | **D** | 0.46  (0.40, 0.51) | 1.18  (1.10, 1.27) | **1.65**  **(1.56, 1.76)** | **D** | 0.34  (0.30, 0.40) | 1.31  (1.21, 1.41) | **1.65**  **(1.56, 1.77)** |
|  |  | **Total** | **4.35**  **(4.20, 4.53)** | **4.57**  **(4.42, 4.72)** | **8.92**  **(8.85, 9.01)** | **Total** | **3.65**  **(3.46, 3.83)** | **5.34**  **(5.20, 5.53)** | **8.99**  **(8.93, 9.08)** |
|  | **Male** |  | **MF** | **M** | **Total** |  | **MF** | **M** | **Total** |
|  |  | **DF** | 2.83  (2.67, 2.99) | 4.22  (4.06, 4.38) | **7.05**  **(6.90, 7.17)** | **DF** | 2.56  (2.38, 2.75) | 4.84  (4.65, 5.02) | **7.40**  **(7.27,7.53)** |
|  |  | **D** | 0.28  (0.24, 0.33) | 1.03  (0.96, 1.10) | **1.31**  **(1.23, 1.40)** | **D** | 0.25  (0.20, 0.31) | 0.99  (0.92, 1.09) | **1.24**  **(1.16, 1.36)** |
|  |  | **Total** | **3.11**  **(2.95, 3.29)** | **5.25**  **(5.08, 5.40)** | **8.36**  **(8.23, 8.46)** | **Total** | **2.81**  **(2.64, 3.02)** | **5.84**  **(5.64, 6.03)** | **8.64**  **(8.57, 8.37)** |
| **80-89** | **Cohort**  **Sex** | 1914-1923 | | | | 1924-1933 | | | |
|  | **Female** |  | **MF** | **M** | **Total** |  | **MF** | **M** | **Total** |
|  |  | **DF** | 2.43  (2.28, 2.57) | 2.66  (2.52, 2.82) | **5.09**  **(4.94, 5.24)** | **DF** | 2.04  (1.86, 2.17) | 3.12  (2.96, 3.28) | **5.15**  **(4.96, 5.30)** |
|  |  | **D** | 0.67  (0.61, 0.76) | 1.78  (1.68, 1.88) | **2.45**  **(2.33, 2.57)** | **D** | 0.49  (0.41, 0.55) | 1.95  (1.83, 2.07) | **2.43**  **(2.29, 2.57)** |
|  |  | **Total** | **3.10**  **(2.93, 3.27)** | **4.41**  **(4.28, 4.62)** | **7.54**  **(7.42, 7.67)** | **Total** | **2.52**  **(2.33, 2.68)** | **5.07**  **(4.87, 5.24)** | **7.59**  **(7.45, 7.70)** |
|  | **Male** |  | **MF** | **M** | **Total** |  | **MF** | **M** | **Total** |
|  |  | **DF** | 1.55  (1.39, 1.71) | 3.49  (3.31, 3.67) | **5.04**  **(4.85, 5.22)** | **DF** | 1.13  (1.00, 1.27) | 3.80  (3.59, 3.98) | **4.93**  **(4.75, 5.11)** |
|  |  | **D** | 0.28  (0.23, 0.33) | 1.41  (1.31, 1.52) | **1.69**  **(1.57, 1.81)** | **D** | 0.28  (0.23, 0.34) | 1.61  (1.51, 1.77) | **1.89**  **(1.76, 2.05)** |
|  |  | **Total** | **1.83**  **(1.65, 2.00)** | **4.90**  **(4.69, 5.08)** | **6.73**  **(6.54, 6.87)** | **Total** | **1.41**  **(1.26, 1.56)** | **5.41**  **(5.23, 5.61)** | **6.82**  **(6.98, 6.73)** |

*Source*: Authors’ calculations based on the HRS

**Table S2.** Partial cohort health expectancies by level of education

| **Ages** |  | **Early Cohort** | | | | **Later Cohort** | | | |
| --- | --- | --- | --- | --- | --- | --- | --- | --- | --- |
| **60-69** | **Cohort**  **Edu** | 1934-1943 | | | | 1944-1953 | | | |
|  | **<HS** |  | **MF** | **M** | **Total** |  | **MF** | **M** | **Total** |
|  |  | **DF** | 3.96  (3.73, 4.14) | 3.09  (2.94, 3.29) | **7.05**  **(6.86, 7.22)** | **DF)** | 2.98  (2.62, 3.39) | 3.60  (3.21, 3.93) | **6.59**  **(6.27, 6.91)** |
|  |  | **D** | 0.60  (0.51, 0.71) | 1.40  (1.27, 1.51) | **2.00**  **(1.85, 2.14)** | Disabled **(D)** | 0.65  (0.48, 0.83) | 1.88  (1.59, 2.12) | **2.53**  **(2.21, 2.83)** |
|  |  | **Total** | **4.55**  **(4.31, 4.77)** | **4.50**  **(4.31, 4.71)** | **9.05**  **(8.94, 9.13)** | **Total** | **3.63**  **(3.24, 4.05)** | **5.49**  **(5.02, 5.87)** | **9.12**  **(8.93, 9.24)** |
|  | **HS** |  | **MF** | **M** | **Total** |  | **MF** | **M** | **Total** |
|  |  | **DF** | 5.15  (5.00, 5.31) | 3.20  (3.08, 3.34) | **8.35**  **(8.26, 8.45)** | **DF** | 4.27  (4.06, 4.45) | 3.99  (3.82, 4.19) | **8.27**  **(8.15, 8.38)** |
|  |  | **D** | 0.36  (0.33, 0.41) | 0.68  (0.62, 0.74) | **1.05**  **(0.97, 1.11)** | **D** | 0.35  (0.30, 0.41) | 0.86  (0.78, 0.95) | **1.21**  **(1.12, 1.31)** |
|  |  | **Total** | **5.51**  **(5.36, 5.67)** | **3.88**  **(3.75, 4.02)** | **9.39**  **(9.34, 9.45)** | **Total** | **4.63**  **(4.42, 4.82)** | **4.85**  **(4.67, 5.07)** | **9.48**  **(9.42, 9.54)** |
|  | **>HS** |  | **MF** | **M** | **Total** |  | **MF** | **M** | **Total** |
|  |  | **DF** | 5.93  (5.68, 6.17) | 3.05  (2.86, 3.29) | **8.99**  **(8.85, 9.10)** | **DF** | 5.67  (5.37, 5.93) | 3.53  (3.30, 3.82) | **9.20**  **(9.07, 9.33)** |
|  |  | **D** | 0.25  (0.20, 0.33) | 0.37  (0.30, 0.43) | **0.63**  **(0.53, 0.73)** | **D** | 0.19  (0.14, 0.23) | 0.36  (0.27, 0.45) | **0.54**  **(0.43, 0.64)** |
|  |  | **Total** | **6.19**  **(5.93, 6.42)** | **3.43**  **(3.20, 3.66)** | **9.61**  **(9.54, 9.67)** | **Total** | **5.86**  **(5.55, 6.12)** | **3.89**  **(3.65, 4.21)** | **9.74**  **(9.68, 9.80)** |
| **70-79** | **Cohort**  **Edu** | 1924-1933 | | | | 1934-1943 | | | |
|  | **<HS** |  | **MF** | **M** | **Total** |  | **MF** | **M** | **Total** |
|  |  | **DF** | 2.69  (2.48, 2.89) | 3.54  (3.36, 3.71) | **6.24**  **(6.06, 6.41)** | **DF** | 2.00  (1.80, 2.22) | 3.97  (3.73, 4.20) | **5.97**  **(5.75, 6.21)** |
|  |  | **D** | 0.43  (0.36, 0.49) | 1.62  (1.50, 1.73) | **2.05**  **(1.91, 2.17)** | **D** | 0.43  (0.33, 0.52) | 1.96  (1.78, 2.11) | **2.39**  **(2.19, 2.58)** |
|  |  | **Total** | **3.12**  **(2.92, 3.32)** | **5.17**  **(4.96, 5.33)** | **8.29**  **(8.15, 8.39)** | **Total** | **2.43**  **(2.18, 2.67)** | **5.93**  **(5.68, 6.16)** | **8.36**  **(8.22, 8.48)** |
|  | **HS** |  | **MF** | **M** | **Total** |  | **MF** | **M** | **Total** |
|  |  | **DF** | 3.57  (3.40, 3.72) | 3.84  (3.69, 3.99) | **7.41**  **(7.28, 7.53)** | **DF** | 3.03  (2.88, 3.20) | 4.49  (4.34, 4.65) | **7.53**  **(7.42,7.64)** |
|  |  | **D** | 0.35  (0.31, 0.41) | 0.99  (0.92, 1.07) | **1.34**  **(1.27, 1.44)** | **D** | 0.28  (0.24, 0.32) | 1.07  (0.99, 1.15) | **1.36**  **(1.27, 1.44)** |
|  |  | **Total** | **3.92**  **(3.75, 4.08)** | **4.83**  **(4.68, 4.99)** | **8.76**  **(8.67, 8.84)** | **Total** | **3.32**  **(3.14, 3.49)** | **5.56**  **(5.42, 5.73)** | **8.88**  **(8.80, 8.95)** |
|  | **>HS** |  | **MF** | **M** | **Total** |  | **MF** | **M** | **Total** |
|  |  | **DF** | 4.05  (3.76, 4.30) | 3.78  (3.55, 4.02) | **7.83**  **(7.64, 8.01)** | **DF** | 3.61  (3.34, 3.90) | 4.61  (4.32, 4.90) | **8.23**  **(8.03, 8.42)** |
|  |  | **D** | 0.40  (0.33, 0.50) | 0.77  (0.65, 0.88) | **1.17**  **(1.04, 1.31)** | **D** | 0.25  (0.19, 0.33) | 0.70  (0.58, 0.82) | **0.95**  **(0.81, 1.08)** |
|  |  | **Total** | **4.45**  **(4.16, 4.70)** | **4.55**  **(4.29, 4.78)** | **9.00**  **(8.88, 9.10)** | **Total** | **3.87**  **(3.57, 4.18)** | **5.31**  **(5.02, 5.60)** | **9.18**  **(9.06, 9.28)** |
| **80-89** | **Cohort**  **Edu** | 1914-1923 | | | | 1924-1933 | | | |
|  | **<HS** |  | **MF** | **M** | **Total** |  | **MF** | **M** | **Total** |
|  |  | **DF** | 1.84  (1.63, 1.98) | 2.63  (2.44, 2.82) | **4.47**  **(4.22, 4.64)** | **DF** | 1.31  (1.13, 1.50) | 2.68  (2.49, 2.92) | **3.99**  **(3.79, 4.25)** |
|  |  | **D** | 0.57  (0.49, 0.67) | 1.85  (1.70, 1.99) | **2.42**  **(2.29, 2.59)** | **D** | 0.45  (0.35, 0.55) | 2.34  (2.12, 2.49) | **2.79**  **(2.56, 2.93)** |
|  |  | **Total** | **2.41**  **(2.17, 2.59)** | **4.48**  **(4.26, 4.67)** | **6.89**  **(6.67, 7.03)** | **Total** | **1.76**  **(1.55, 1.98)** | **5.02**  **(4.77, 5.22)** | **6.78**  **(6.60, 6.92)** |
|  | **HS** |  | **MF** | **M** | **Total** |  | **MF** | **M** | **Total** |
|  |  | **DF** | 2.11  (1.96, 2.26) | 3.11  (2.94, 3.26) | **5.22**  **(5.07, 5.38)** | **DF** | 1.72  (1.55, 1.84) | 3.60  (3.41, 3.75) | **5.32**  **(5.13, 5.46)** |
|  |  | **D** | 0.51  (0.45, 0.59) | 1.58  (1.48, 1.67) | **2.09**  **(1.98, 2.20)** | **D** | 0.36  (0.30, 0.43) | 1.69  (1.58, 1.83) | **2.06**  **(1.95, 2.20)** |
|  |  | **Total** | **2.62**  **(2.45, 2.80)** | **4.69**  **(4.50, 4.86)** | **7.31**  **(7.17, 7.44)** | **Total** | **2.09**  **(1.89, 2.23)** | **5.30**  **(5.12, 5.47)** | **7.38**  **(7.25, 7.49)** |
|  | **>HS** |  | **MF** | **M** | **Total** |  | **MF** | **M** | **Total** |
|  |  | **DF** | 2.50  (2.18, 2.89) | 3.42  (3.10, 3.78) | **5.92**  **(5.61, 6.29)** | **DF** | 1.98  (1.70, 2.29) | 3.74  (3.44, 4.05) | **5.71**  **(5.47, 6.01)** |
|  |  | **D** | 0.37  (0.26, 0.47) | 1.37  (1.14, 1.52) | **1.74**  **(1.48, 1.90)** | **D** | 0.45  (0.35, 0.56) | 1.49  (1.28, 1.69) | **1.94**  **(1.73, 2.15)** |
|  |  | **Total** | **2.86**  **(2.50, 3.27)** | **4.79**  **(4.39, 5.12)** | **7.65**  **(7.41, 7.90)** | **Total** | **2.42**  **(2.10, 2.77)** | **5.23**  **(4.89, 5.55)** | **7.65**  **(7.49, 7.85)** |

*Source*: Authors’ calculations based on the HRS
